# A Novel PACS1 Variant Associated With Schuurs-Hoeijmakers Syndrome Phenotype in an Indigenous Descendant in Brazil: A Case Report

**DOI:** 10.7759/cureus.30486

**Published:** 2022-10-19

**Authors:** Pedro H Lucena, Carolina Nonaka, Giulia Armani-Franceschi, Pedro Carneiro, Henrique Sales, Mariana Lucena, Igor D Bandeira, Bruno Solano, Rita Lucena

**Affiliations:** 1 Instituto da Criança, Universidade de São Paulo, São Paulo, BRA; 2 Instituto D’Or para Pesquisa e Educação, Centro de Biotecnologia e Terapia Celular, Salvador, BRA; 3 Faculdade de Medicina da Bahia, Universidade Federal da Bahia, Salvador, BRA; 4 Department of Psychiatry and Behavioral Sciences, Stanford University, Stanford, USA; 5 Instituto Gonçalo Moniz, Fundação Oswaldo Cruz, Salvador, BRA; 6 Departamento de Neurociências e Saúde Mental, Universidade Federal da Bahia, Salvador, BRA

**Keywords:** schuur-hoeijmakers’ syndrome, pacs1, neurodevelopmental delay, autism spectrum disorder (asd), intellectual disability (id)

## Abstract

Schuurs-Hoeijmakers syndrome, an autosomal dominant disorder associated with mutations in the PACS1 gene, was initially identified in two unrelated children of European descent from a cohort of individuals with intellectual disabilities. This gene alteration significantly reduced cranial cartilaginous structures, inducing craniofacial alterations predominantly in a dominant-negative fashion. In this paper, we report a novel variant of PACS1 associated with Schuurs-Hoeijmakers syndrome: a boy aged two years and nine months of indigenous descent presenting with motor stereotypies, atypical sensory searches, language delay, and low socio-interactional reciprocity. Whole exome sequencing confirmed the presence of a heterozygous missense mutation c.943C>T p. (Arg315Trp) in the PACS1 gene. The phenotypic profile identified was similar to the other cases of Schuurs-Hoeijmakers syndrome described in the literature. This report highlights the importance of considering the possibility of PACS1 gene alterations and a diagnosis of Schuurs-Hoeijmakers syndrome in patients presenting craniofacial alterations associated with autistic features, psychomotor and language development delay.

## Introduction

Genomic sequencing techniques are now regularly used in clinical practice. This includes searching for genetic variants associated with intellectual disabilities and has facilitated the identification of rare diseases and their associated gene variants among populations with different ethnic compositions, and the impact of these variants on neurodevelopment is a growing field of study.

In this context, Schuurs-Hoeijmakers syndrome (SHS), an autosomal dominant disorder characterized by delayed intellectual developmental and craniofacial and/or congenital abnormalities, was first discovered in two unrelated children of European origin in a cohort study of over 5000 individuals with intellectual disabilities [1]. In addition to presenting significant phenotypic similarities, whole-exome sequencing showed that children affected with Schuurs-Hoeijmakers syndrome presented with mutations in the PACS1 gene [1]. The PACS1 mRNA expression is abundant during embryonic brain development, while the PACS1 protein regulates the trans-membrane transport of the Golgi complex and the endosome-to-Golgi pathway [2]. Mutations in the PACS1 gene can lead to the migration of defective neural crest cells, which lead to morphological changes in the skull and facial tissue [1,2].

In 2015, Gadzicki and colleagues reported a third case of Schuurs-Hoeijmakers syndrome [3] and a year later, Schuurs-Hoeijmakers et al. reported phenotypic characteristics in 19 subjects with the PACS1 mutation, involving 16 new cases [4]. Current prevalence of the syndrome is largely unknown, with around 60 cases reported in the literature [4-13], although genetic penetrance appears to be 100% [14]. To the best of our knowledge, this is the second reported case of Schuurs-Hoeijmakers syndrome to be identified in Brazil and the first that affects an indigenous descendant.

## Case presentation

A boy of two years and nine months of indigenous descent and presenting developmental delay was referred for neurological evaluation. Obstetric history did not reveal anything other than a urinary tract infection successfully treated with cephalexin. He was born at term, by vaginal delivery, and was healthy, weighing 3.11 kg. He presented early neonatal jaundice with maximum indirect bilirubin of 11 mg/dL, which cleared after two days of phototherapy. He is the only child of a non-consanguineous couple, whose father died of a stroke while being treated for bowel cancer. 

The child acquired head control at two months, was able to roll completely at six months, sit with support at 18 months, and unsupported at 30 months. Before completing his first year, motor stereotypes and atypical sensory searches such as placing objects in the mouth, rolling from side to side, and hand flapping became apparent. Language delay and poor reciprocal social interaction were detected at 12 months. During clinical evaluation, he was still unable to point or reproduce simple gestures and vocalizations presented low phono-articulatory diversity and were devoid of communicative intent. Mild progress was observed during physical therapy, speech therapy, and occupational therapy, with no regression of neurodevelopment.

The neurological evaluation suggested global developmental delay, with the cognitive performance of an eight-month-old baby (Bayley III). The child presented good tactile, auditory and visual localization reactions, with accentuated sensory seeking, including oral experimentation with objects, and repetitive and dysfunctional movements. Hypotonia and ligament laxity were present, although there was no evidence of muscle strength deficit. Motor development delay was accompanied by cognitive impairment, autistic manifestations, and global dyspraxia. There was an inability to modulate strength and precision of movements, but there were no associated signs of pyramidal release, involuntary movements, or ataxias. Deep reflexes were globally diminished with no evidence of peripheral nervous system impairment. Craniofacial alterations such as full and arched eyebrows, a bulbous nasal tip and thin upper lip were also noted. 

While initial hematological and biochemical assessments were considered normal, at two years and seven months, maximum leukocytosis levels were detected during an acute, nonspecific febrile illness: 52,100/mm3 leukocytes with 13% atypical lymphocytes, decreasing to 24,700 and 24%, respectively, after two days. Spherocytosis was diagnosed, and osmotic fragility performed. Leukocytosis was a secondary result of hemolytic anemia, due to spherocytosis. Alkaline phosphatase levels were excessive (571 U/L / RV: 104-345 U/L), but showed no clinical signs of cholestasis and the abdominal ultrasound result was normal. 

Magnetic resonance imaging revealed mild ectasia of the lateral ventricles while the karyotype revealed heteromorphism and was considered a variant of normality (arr (1-22) x2, (xy), x1). Array comparative genomic hybridization (CGH) analysis did not reveal any pathogenic copy number variation.

Informed consent was obtained from the family and the Institutional Review Board (IRB) at the Professor Edgard Santos University Hospital, Salvador, Bahia, Brazil gave approval 4402125. A single proband whole exome sequencing was performed to investigate a possible genetic cause for the disorder. Exon capture was performed by extracting leukocytes from peripheral blood and more than 1700 genes were then sequenced using next generation sequencing (NGS) on the Illumina system (NGS Twist Human Core Exome Plus kit, Twist Bioscience HQ, San Francisco, CA, USA). Analysis involved 99% of target bases with at least ≥10x and ≥20x of vertical coverage, while 95% covered ≥50x. Alignment and identification of variants was carried out using the human genome (UCSC Genome Browser GRCh37/hg19) as a reference. Annotation, filtering, and variant prioritization were performed using internal bioinformatics pipelines and Human Gene Mutation Database (HGMD®), ClinVar (clinically significant variants), and minor allele frequency (MAF). Strict quality criteria and validation processes were established for variants detected by NGS. Sanger sequencing subsequently confirmed low-quality single nucleotide variants or relevant insertion and/or deletion variants.

**Figure 1:**
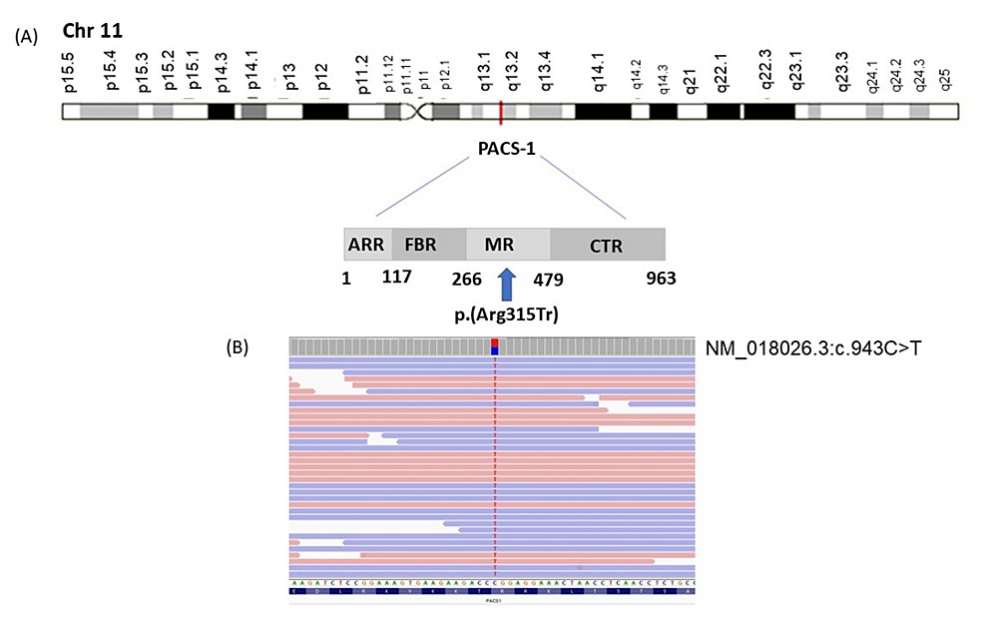
Localization of the PACS1 variant identified (A) Schematic diagram of location and protein structure of PACS-1 showing the atrophin-related region (ARR), the furin-binding region (FBR), the middle region (MR), and the C-terminal region (CTR). The arrow indicates the p.Arg315Tr in the autoregulatory domain (MR) of the protein. (B) Schematic NM_018026.3:c.943C>T location by RefSeq (www.ncbi.nlm.nih.gov/refseq)

Whole exome sequencing analysis revealed a heterozygous missense mutation in the PACS1 gene at Chr11:66216740 (hg19), NM_018026.3:c.943C>T p.(Arg315Tr), single nucleotide polymorphism (SNP) identifier rs371513573, CCDS8129.1, and was classified as a variant of uncertain significance (VUS) (Figure 1). This missense variant is absent from gnomAD, ABraOM, and is considered deleterious by different silico tools, including Polyphen-2, PROVEAN, SIFT, and Mutation Taster. The variant leads to changes in the amino acid sequence and alterations in a splice site. The Online Mendelian Inheritance in Man (OMIM) phenotype number is #615009 and indicates autosomal dominant mental retardation 17 (MRD17) caused by heterozygous mutation in the PACS1 gene. The variant was not found in gnomAD, and it was not reported in the ClinVar database. The suggested classification for the variant according to the American College of Medical Genetics is PM2, PP3, PP2. Table 1 highlights previous case reports with similar mutations.

**Table 1:** Previous case reports with similar mutations

Phenotype	Current Report	Schuur-Hoeijmakers et al. (2016) [4]	Stern et al. (2017) [5]	Miyaki et al. (2017) [6]	Martinez-Monseny et al. (2018) [7]	Hoshino et al. (2018) [8]	Pefkianaki et al. (2018) [9]	Dutta et al. (2019) [10]	Colak et al. (2020) [11]
Number of subjects	1	19	8	1	1	2	1	1	1
Gender	Male	7 F and 12M	2 F and 6M	Female	Female	1 F and 1M	Female	Male	Male
PACS1 (NM_018026.3) variants	c.943C>T p.(Arg315Tr)	c.607C>T p(Arg203Trp)	c.607C>T p(Arg203Trp)	c.608G>A, p.(Arg203Gln)	c.607C>T p(Arg203Trp)	c.607C > T (p.Arg203Trp)	c.607C > T (p.Arg203Trp)	c.607C > T (p.Arg203Trp)	c.607C > T (p.Arg203Trp)
Flat philtrum	-	+	+ (1/8)	-	+	-	-	-	-
Low-set ears	-	+	+ (1/8)	-	+	+	-	+	-
Full, Arched eyebrows	+	+	+ (2/8)	+	-	+	+	-	+
Long eyelashes	-	+	+ (1/8)	-	-	+	-	+	+
Hypertelorism	-	+	+ (3/8)	+	+	+	-	+	+
Downslanting palpebral fissures	-	+	+ (3/8)	-	+	+	+	-	-
Ptosis	-	+	+ (1/8)	-	-	-	-	-	+
Nystagmus	-	+ (2/19)	-	-	-	-	-	-	-
Strabismus	-	+ (2/19)	-	-	-	-	-	-	-
Myopia	-	+ (5/19)	-	-	-	-	+	-	-
Bulbous nasal tip	+	+	+ (4/8)	-	+	+	+	+	+
Wide mouth	-	+	+ (1/8)	-	+	+	+	+	+
Thin upper lip	+	+	+ (1/8)	-	+	+	+	+	+
Oromotor sensitivity	-	+ (11/19)	-	-	-	-	-	-	+
Downturned corners of the mouth	-	+	+ (1/8)	-	+	+	+	-	+
Cardiovascular malformation	-	+ (10/19)	+ (4/8)	-	+	+	-	-	+
Feeding difficulties	-	+ (5/19)	-	-	-	+	-	-	+
Gastric reflux	-	+ (6/19)	-	-	-	-	+	-	-
Constipation	-	+ (9/19)	-	-	-	+	+	-	-
Cryptorchidism	-	+ (6/12)	-	-	-	+	-	-	+
Pes planus	+	+ (4/19)	-	-	-	-	+	-	-
Hypotonia	+	+ (8/19)	-	+	-	+	-	-	-
Delayed psychomotr development	+	+	+	+	+	+	+	+	+
Intellectual disability	+	+	+	+	+	+	+	+	+
Language delay	+	+	+	-	+	+	+	+	+
Seizures	-	+ (12/19)	+ (5/7)	-	+	+	-	-	-
Cerebellar hypoplasia	-	+ (4/16)	-	-	-	-	-	-	+
Ventricular abnormalities	+	+ (4/16)	-	-	-	-	-	-	+
White matter defects	-	+ (3/16)	-	-	-	-	-	-	+
Aggressive behavior	-	+ (10/19)	-	-	-	-	-	-	-
Autistic features	+	+ (6/19)	+ (2/7)	-	-	-	+	+	-
Laughing episodes	-	-	-	-	+	-	-	-	-
Note: (+) indicates the presence of the clinical feature; (−) clinical feature not reported.									

## Discussion

This is the first reported Schuurs-Hoeijmakers case associated with a novel PACS1 variant. PACS1 is a regulator of protein transport across the Golgi membrane. mRNA expression is higher during the embryonic brain development period and lower after birth [15]. Extensive functional studies have shown that an altered PACS1 expression in zebrafish embryos promotes a significant reduction in cranial cartilage structures, inducing craniofacial defects predominantly in a dominant-negative fashion. The phenotype was attributed to the atypical specification and migration of SOX10-positive cells in the neuronal crest, which compromises the migration of cells along the brachial arch [1]. 

Schuurs-Hoeijmakers syndrome is characterized by pathogenic variants in the PACS1 gene leading to a phenotype characterized by intellectual developmental delay, craniofacial abnormalities, and variable congenital abnormalities. The craniofacial changes reported in this study (thick and arched eyebrows, bulbous nasal tip, and thin upper lip) are not as prominent as the previously described cases in the literature. The frequency of such findings reported in the literature ranges from 76.4% to 85.2%. However, functional characteristics such as delayed psychomotor and language development were present in all individuals with the syndrome. Macro and microcephaly [4,6,7,11], scoliosis [4,9], pectus excavatum [4,9], small teeth [5], alterations in the palate [5,10], finger and toe alterations [5,7], short stature, growth impairment [6,7,11] and coloboma [8,10] are also associated with variants of PACS1, though less frequently expressed. Additionally, Silva et al. reported microcornea and microphthalmia as ophthalmological findings related to SHS [16].

The child was diagnosed with hereditary spherocytosis (HS) during an investigation into leukocytosis that was dissociated from infectious events. Up to that point, there had been no reports of hematological changes in subjects with Schuurs-Hoeijmakers syndrome. Changes to the proteins spectrin (SPTA1 and SPTB genes), ankyrin (ANK1 gene), band 3 (SLC4A1 gene), and band 4.2 (EPB42 gene) are known to cause changes in the membrane/cytoskeleton of red blood cells, making them fragile. These changes are known causes of HS [17]. Without other gene candidates, it is not possible to establish a causal relationship between HS and PACS1 variation. However, since this is the only reported case of variant c.943C>T (p.Arg315Tr), such an association cannot be excluded.

It is worth noting the present study’s limitations, in that only one patient with the variant was included and the heritability of the variant could not be evaluated since the father had died before carrying out the study. Also, expanding the analysis to cover siblings was not possible as the patient was an only child. Further research will therefore be necessary to confirm the pathogenicity of the PACS1 variant described in this study. 

## Conclusions

This report contributes to the understanding of pathogenicity related to variations of the PACS1 gene. Even though this specific genetic variation had not previously been reported, the phenotypic profile shares similarities with previously described cases. The diagnosis of HS and the lack of other candidate genes suggest that the PACS1 gene may have a broader role within individuals’ phenotype. Craniofacial alterations associated with autistic features, as well as psychomotor and language development delay should raise the possibility of PACS1 alteration, and genetic testing should therefore be performed at an early stage in order to reach a timely diagnosis and provide appropriate counseling to the family.
